# 肺良性转移性平滑肌瘤1例

**DOI:** 10.3779/j.issn.1009-3419.2012.01.12

**Published:** 2012-01-20

**Authors:** 雯月 张, 敏 李, 红忠 杨, 成平 胡

**Affiliations:** 410008 长沙，中南大学湘雅医院呼吸科 Department of Respiratory Medicine, Xiangya Hospital, Center South University, Changsha 410008, China

## 1 病例资料

患者，女，33岁，已婚。因体检发现双肺多发性肿块10个月于2011年5月入住湘雅医院呼吸科。患者无咳嗽、气促、咯血、发热、盗汗、胸痛等症状。既往病史2002年发现“子宫肌瘤”，于2002年和2004年两次行子宫肌瘤剥除术，2010年9月行子宫+双附件切除术，术后病理提示“（子宫）平滑肌瘤”。入院体查：左颈部及骶尾部皮下可分别触及1个4 cm×4 cm和1.5 cm×1.5 cm软组织肿块，边界清，活动度可，有压痛。右侧锁骨上可触及3枚肿大淋巴结，大者约蚕豆大小，质韧，边界清，活动度可，有压痛。胸廓无畸形，无局限性隆起及凹陷，双肺呼吸音清，未闻及干湿啰音。

入院后胸部CT检查（平扫+增强）提示双肺散在大小不等结节影，最大者位于右上肺前段，约5.2 cm× 6.5 cm×7.6 cm大小，病灶边界均清晰、光整、密度尚均匀，双肺门及纵隔见多发大小不等淋巴结（图 1）。B超示盆底低回声肿块（69 cm×33 cm×50 mm）性质待定，盆腔偏左囊性结节（31 cm×30 cm×30 mm）性质待定。血FSH、LH、PRL、雌二醇、孕酮、睾酮、β-HCG均正常。左颈部肿块病检结果为左颈部梭形细胞肿瘤，考虑平滑肌瘤（图 2）；免疫组化示：平滑肌Actin（-），Bc1-2（+），CD117（-），血管CD34（+），CD99（-），CK-Pan（-），Desmin（+），Dog-1（-），EMA（-），血管F8（+），HHF35（+），Nestin（+），S-100（-），Vimentin（+）。右上肺肿块CT引导下穿刺活检（图 3）示右上肺梭形细胞肿瘤，考虑平滑肌源性肿瘤，未见坏死，核分裂相0-2/10HPF，细胞异型性不大，结合病史，倾向良性转移性平滑肌瘤；免疫组化：CD34（-），CD117（-），Dog-1（-），S-100（-），SMA（+），Bc1-2（-），CD99（±），Desmin（+）。患者出院后一直坚持孕激素治疗，并于当地医院完善右锁骨上淋巴结及骶尾部肿块活检，病理亦提示平滑肌瘤。2011年10月患者在当地医院接受伽马刀治疗，目前仍在随访中。

**1:**
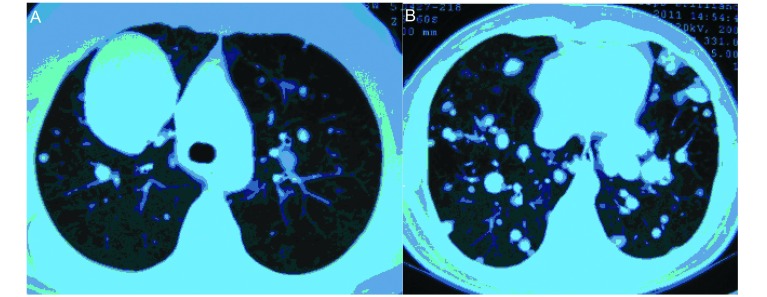
胸部CT。A：右上肺前段可见一大小约5.2 cm× 6.5 cm×7.6 cm的肿块；B：双肺散在大小不等结节影，病灶边界均清晰、光整、密度尚均匀。 Chest CT. A: a mass about 5.2 cm×6.5 cm×7.6 cm in the anterior lobe of right upper lung; B: lung nodules of varying sizes, with clear boundaries, smooth fringes, and uniform density.

**2:**
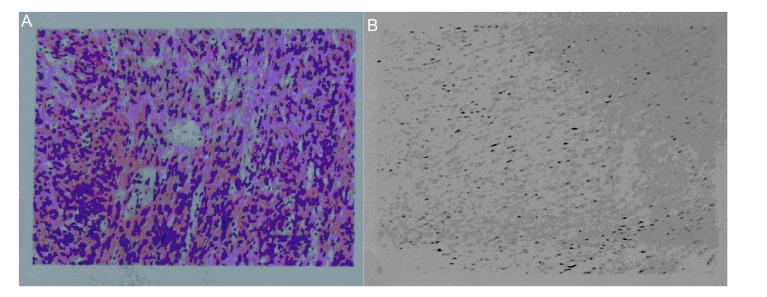
左颈部肿块病检。A：梭形细胞瘤，瘤细胞呈梭形状，呈束状，交织状，漩涡状排列，胞浆呈嗜酸性，细胞核染色质呈细沙状（HE, ×200）。B：梭形瘤细胞，HHF35阳性（免疫组化，×200）。 Pathological examination of mass in left neck. A: Spindle cell tumor, tumor cells were spindle shaped, and arranged like bundles, wovening, or swirling. The cytoplasm was eosinophilic, and chromatin was fine sand-like (HE, ×200). B: Spindle-shaped tumor cells, HHF35 positive (Immunohistochemistry, ×200).

**3:**
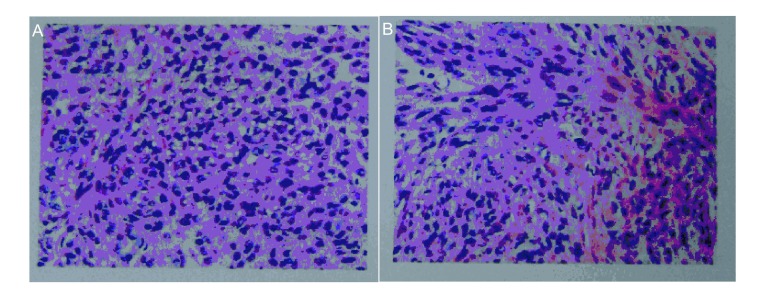
右上肺肿块病检。A：梭形细胞瘤，呈束状排列，细胞丰富，胞浆嗜酸性（HE, ×400）。B：梭形瘤细胞，可见部分核分裂像，核异型性不大（HE, ×400）。 Pathologic examination of mass in right upper lung. A: spindle cell tumor, which were arranged in bundles, rich in cells, and eosinophilic cytoplasm (HE, ×400). B: spindle-shaped tumor cells, showing part of mitotic, and mild nuclear atypia (HE, ×400).

## 2 讨论

肺良性转移性平滑肌瘤（pulmonary benign metastasizing leiomyomatosis, PBML）是一种罕见的疾病。一般认为良性肿瘤无转移性，但Steiner等^[1]^却在1939年首先报道了肺良性转移性平滑肌瘤。PBML通常是指有子宫平滑肌瘤病史的患者在肺部出现转移性，但其它生物学表现为良性的平滑肌肿瘤^[2]^。随着研究的深入，这一疾病逐渐被人们了解和认识，但其病理起源在不同研究报道中仍存争议，主要有3种假说：①肿瘤来源于转移的良性子宫平滑肌瘤^[2]^，此为主流学说；②肿瘤转移自低度恶性的平滑肌肉瘤^[3]^；③多中心生长的平滑肌瘤，此学说认为该瘤应为原发于肺的多发性平滑肌瘤性错构瘤（multiple pulmonary leiomyomatous hamartoma, MPLH）或多发性纤维平滑肌瘤性错构瘤（multiple pulmonary fibroleiomyomatous hamartoma, MPFLH）^[4]^。

1977年Horstmann等^[2]^将平滑肌瘤性错构瘤、纤维平滑肌性错构瘤等改称为良性转移性平滑肌瘤。PBML多发生于育龄妇女，大部分患者有子宫肌瘤史及部分子宫、全子宫切除的手术史^[5]^。在子宫以外的部位，如肺、主动脉旁淋巴结、四肢的横纹肌、皮肤及瘢痕等处出现组织学良性的平滑肌瘤，其中以肺多见^[3, 4]^。肺部病变多出现在子宫术后3个月-20年。

PBML临床过程缓慢，大部分患者无自觉症状，常常是体检中无意发现，少数患者可有发热、干咳、咯血、胸闷、气促等。Horstmann等^[2]^回顾了23例PBML的影像学表现，其中最常见的影像学表现（16例，70%）为双肺内的多发结节，结节边缘较光滑，可有分叶，也可出现空洞；其次为单侧多发肿块（4例，17%），而单发肿块则最少（3例，13%），罕见表现为粟粒样弥漫性病变。PBML的确诊有赖于病理检查。在组织学方面，确定其原发灶（子宫平滑肌瘤）细胞核形态、分裂相无肉瘤（恶性）样变，同时肺内或其它器官的转移结节病理与之相似。在免疫组化方面，所有的瘤体均来源于间叶组织，激素受体情况也相似。在良性病变确定后，从行为学上才能认定其转移之特性^[3]^。临床上PBML需与转移瘤、淋巴管平滑肌瘤病、平滑肌肉瘤、炎性假瘤等相鉴别。

本例为育龄期女性患者，既往有子宫肌瘤病史多年，先后行子宫肌瘤剥除术2次以及子宫+双附件切除术，术后病理证实为“平滑肌瘤”。患者无咳嗽、咯血、气促等不适，于体检时发现双肺多发结节影，病灶边界均清晰、光整、密度尚均匀，门诊随诊期间发现患者肺部结节影增多、变大，且在左颈部、骶尾部皮下等多处发现肿块。我院及外院多处病变部位活检均证实为平滑肌源性肿瘤，且核分裂少，细胞异型性不大，排除恶性病变可能，因此肺良性转移性平滑肌瘤诊断成立。

目前对于PBML的治疗方案为：①首选外科手术切除，对于能够手术切除的病灶应予切除，手术切除后的患者应随诊，密切观察肺内有无新病灶出现；②外科卵巢切除或药物性闭经，该方案主要通过阻断激素的释放来稳定肺内病变^[6]^；③下腔静脉滤网植入，有报道^[7]^指出腹部手术后放置下腔静脉过滤网对预防原发病灶转移至肺部有很好的疗效。Kayser等^[8]^报道的10例良性转移性平滑肌瘤在行手术切除后中位生存时间为94个月，而同时观察的2例转移性平滑肌肉瘤最长生存期为22个月。

本文报道的此例患者肺内病灶多，散在分布，无法手术治疗；且患者在双侧卵巢切除术后1年发现肺内多发病变，虽血雌激素水平在正常范围内，但肺内病灶仍呈慢性进展趋势，以上信息均提示预后不良。患者目前在接受孕激素治疗，但疗效仍有待随访。

虽然PBML在我国罕见，报道病例为数不多，但临床医师仍应重视。如临床上遇到育龄期女性、有子宫肌瘤病史、肺内出现结节或弥漫性病变，需考虑PBML可能，并尽可能对多个病变部位行活检术，明确病理类型及来源。对能手术切除的病例，应首选手术治疗，且应在术后随访监测患者性激素水平和肺内病变变化情况。
